# Omics Integration Analysis Unravel the Landscape of Driving Mechanisms of Colorectal Cancer

**DOI:** 10.31557/APJCP.2020.21.12.3539

**Published:** 2020-12

**Authors:** Fatemeh Nikmanesh, Shamim Sarhadi, Mehdi Dadashpour, Yazdan Asgari, Nosratollah Zarghami

**Affiliations:** 1 *Stem Cell Research Center, Tabriz University of Medical Sciences, Tabriz, Iran. *; 2 *Department of Medical Biotechnology, Faculty of Advanced Medical Sciences, Tabriz University of Medical Sciences, Tabriz, Iran. *; 3 *Iranian Blood Transfusion Organization-Research Center, Iranian Blood Transfusion Organization, IBTO blg., Hemmat Exp. Way, Teheran, Iran. *; 4 *Department of Medical Biotechnology, School of Advanced Technologies in Medicine, Tehran University of Medical Sciences, Tehran, Iran. *; 5 *Department of Clinical Biochemistry and Laboratory Medicine, Faculty of Medicine, Tabriz University of Medical Sciences, Tabriz, Iran. *

**Keywords:** Colorectal cancer, flux balance analysis, omics integration, regulome

## Abstract

Colorectal cancer (CRC) is one of the most malignant cancers and results in a substantial rate of morbidity and mortality. Diagnosis of this malignancy in early stages increases the chance of effective treatment. High-throughput data analyses reveal omics signatures and also provide the possibility of developing computational models for early detection of this disease. Such models would be able to use as complementary tools for early detection of different types of cancers including CRC. In this study, using gene expression data, the Flux balance analysis (FBA) applied to decode metabolic fluxes in cancer and normal cells. Moreover, transcriptome and genome analyses revealed driver agents of CRC in a biological network scheme. By applying comprehensive publicly available data from TCGA, different aspect of CRC regulome including the regulatory effect of gene expression, methylation, microRNA, copy number aberration and point mutation profile over protein levels investigated and the results provide a regulatory picture underlying CRC. Compiling omics profiles indicated snapshots of changes in different omics levels and flux rate of CRC. In conclusion, considering obtained CRC signatures and their role in biological operating systems of cells, the results suggest reliable driver regulatory modules that could potentially serve as biomarkers and therapeutic targets and furthermore expand our understanding of driving mechanisms of this disease.

## Introduction

Cancer is the leading cause of death worldwide (Rasouli et al., 2020). There are several type of cancer, such as lung, stomach, colorectal, liver, and breast (Sheervalilou et al., 2016; Maasomi et al., 2017; Sadeghzadeh et al., 2017). Among the various cancers, gastrointestinal cancers occur mainly in men and women living in developing countries (Lotfi-Attari et al., 2017, Mohammadian et al., 2016a; Mohammadian et al., 2016b).

Colorectal cancer (CRC) is the third most common cancer among men and women in the US and affects all racial groups (Boyle and Ferlay, 2005; Siegel et al., 2016). The reports indicated 50,260 deaths during 2017 (Siegel et al., 2016; Siegel et al., 2017). Similar to other types of cancers, CRC could be prevented and treated with higher probability if detection occurs at the early stage of tumor initiation (Levin et al., 2008). There are several common tests to detect this malignancy including colonoscopy and serum Carcinoembryonic Antigen (CEA) test, but their sensitivity is not desirable (Zou et al., 2015; Sheervalilou et al., 2016; Maasomi et al., 2017; Sadeghzadeh et al., 2017). There are also some common methods to screen susceptible individuals such as endoscopic, histopathological examination of biopsies, and surgically removed specimens that are undesirable in terms of sensitivity and also are painful which bring complications for patients (Zheng et al., 2013). Generating biological high-throughput data and optimization of computational methods obviate obstacles to study biological systems using systems biology as a multidisciplinary approach (Ideker et al., 2001). While omics puzzle is being completed in parallel with evolving data integration and computational technics (Machado and Herrgård, 2014), numerous studies have tried to find biomarkers and enhanced the chance of early detection to improve diagnosis and prognosis of patients (Levin et al., 2008; Huang et al., 2010; Vatandoost et al., 2016). Since in an up to bottom approach in omics levels, metabolome perturbation is the final change, it could provide a clearer vision of cell abnormalities status during initiation and progression of disease due to alteration in genomic, post-genomic, transcriptomic, and proteomic profiles (O’Connell, 2012; Zhang et al., 2014). Besides, it has been shown that metabolic perturbation is one of the common characteristics of cancerous cells. Numerous studies have tried to develop metabolic models by integrating gene expression data in the metabolic framework of target cells and design predictive cancerous metabolic models which could help to understand differences between tumor and normal samples (Hagland and Søreide, 2015; Mika et al., 2017). Exploring such alterations in genome, transcriptome, and metabolome could pave the way of finding biomarkers and understanding metabolic alterations that may be useful for diagnosis at the early stage of cancer. Study of underlying molecular mechanisms of CRC has conducted in many research by systems biology approach (Madhavan et al., 2013). Accumulating changes in CNA, point mutations, gene expression and flux balance alteration of metabolic drivers indicated to promote the cancer initiation and progression (Corti et al., 2019). In this study, we tried to unravel the landscape of metabolic, genomic and transcriptomic alterations and their integrated role in fine-tuning of regulome for initiation and progression of CRC by systems biology approach and using the huge numbers of publicly available data. Since there have been very few studies that simultaneously considered changes in different omics levels for CRC, in this study we applied data from different sources to obtain signatures of each omics levels that could be applied to understand the molecular mechanisms of CRC. 

## Materials and Methods

*Data collection*

Gene expression microarray data related to colorectal cancer were downloaded from the Gene Expression Omnibus (GEO) database. Microarray data platform was Affymetrix Human Genome U133 Plus 2.0 Array (GPL570) for both datasets. To analyzing copy number aberration and point mutations related to CRC, next generation sequencing data retrieved through Cbioportal (Cerami et al., 2012; Gao et al., 2013). Moreover, TCGA data including gene expression, copy number alteration, point mutation, microRNA, methylation, and protein profiles used to portrait regulome signatures of CRC. 

Microarray raw data were preprocessed using MATLAB software (version R2015b). First, a preprocessing of gene expression raw data has been performed by transferring all gene expression values to log2 scale, and by doing normalization step (using quantilenorm command in MATLAB based on Median). This procedure was repeated for both data types (normal and cancer). 

*Metabolic Model Reconstruction*

We have used the corrected human metabolic model (Shlomi et al., 2011) which includes 2766 metabolites, 3748 reactions, and 1905 genes to work with. Gene expression values corresponding to metabolic genes have been mapped into the human metabolic model using E-Flux method (Colijn et al., 2009). E-Flux approach uses pre-processed gene expression data, finds metabolic genes through them, and considers the corresponding expression values. Next, all values were replaced into the gene-reaction association matrix available in the human metabolic model. The new gene-reaction association matrix now includes some changed elements due to mathematical operators and some unchanged elements because they did not have any metabolic genes. Then, the gene-reaction association matrix values were rescaled to [0,1], multiplied by the current upper bound values of the model (values for the human metabolic model), and set as the new upper bound values of the model. The lower bound values were negative values of the new upper bound values (for reversible reactions). Therefore, this method constrains the upper and lower bounds of each reaction according to its corresponding gene expression level. We wrote a Mathematica script to apply E-Flux algorithm to the human metabolic model. So, we have reconstructed normal and cancer colorectal metabolic models. The biomass and the medium compositions (RPMI-1640) added to the models are shown in the supplementary file 1. All normal and cancer metabolic models are also available in the supplementary file 2 as MATLAB structure files.

*Flux Balance Analysis*

Flux Balance Analysis (FBA) considers appropriate constraints whereas the system is in its steady-state. In FBA, a metabolic network is considered as a stoichiometric set of equations (in a matrix format including the stoichiometric (S) and the flux (V) matrices (Masoudi-Nejad and Asgari, 2015). Since a higher number of reactions in comparison to metabolites is a common feature of most metabolic networks, this property prevents the system of linear equations to be solved analytically. One approach is using linear programming in which it tries to solve a system of equations in association with minimization/maximization of an objective function as follows:

min/max: c^T^.*v*

subject to: S.*v* = 0 a < *v* < b

where c^T^ is a transposed vector of stoichiometric coefficients of metabolites incorporating with the objective function, and v is a vector of fluxes which will be determined. Vectors a and b are also lower and upper bounds of all reaction, respectively. The solution of the metabolic fluxes is underdetermined when a system is unconstrained. Applying additional constraints (eg. a < *v* < b) would decrease the solution space. In such a condition, one could obtain the optimal set of the flux distribution while an objective function is optimized. Therefore, FBA would turn into a linear programming (LP) problem. For this study, we used the COBRA toolbox (Constraints Based Reconstruction and Analysis) for evaluating the metabolic models by maximization of the biomass equation (Feist and Palsson, 2010). The glpk solver was used for linear programming problems. For FBA, running the optimizeCbModel function builds four main output structures: f, x, w, and y, where f is the objective value, x includes reaction fluxes, w is a vector of reduced costs, and y is a vector of shadow prices. Every reaction belongs to a known metabolic subsystem. For example, there are 99 subsystems presented in the human metabolic model according to metabolic pathway classification (Lehninger et al., 2005). Subsystems information are available through the vector called subSystems in the MATLAB model structure. All FBA results related to normal and cancer metabolic models are available in the supplementary file3. 

*Differentially expression gene and gene enrichment analysis by GSEA*

To find differentially expressed genes (DEGs) in CRC cells in comparison with the normal cells, gene expression microarray data sets preprocessed by FRMA package (McCall et al., 2010) in R and then limma (Smyth, 2005) was used to develop a linear model for finding DEGs. Obtained DEGs for each dataset combined by fisher method. To find pathways that over and under-expressed in CRC samples mentioned datasets merged by COMBAT method to remove study bias. In the next step, a dataset consisting of DEGs and their expression values and Reactom geneset fed into the GSEA (Subramanian et al., 2007). To run the software, permutation type set to gene-set and the number of permutations set to 1000. After finding enriched pathways, 20 top-ranked pathways selected for leading edge analysis that clusters genes according to the number of genes involved in common gene-sets. 

*Network analysis of genomic and transcriptomic signatures of CRC*

To illustrate tissue-specific networks in genomic and transcriptomic levels of CRC, signatures corresponding to DEGs, point mutations and CNAs were constructed by data retrieved from the DifferentialNet database (Basha et al., 2017). The DEGs network created by DEGs with signal to noise ratio (SNR) more than 0.5. Also, in case of point mutation and CNA signatures frequency of 5% defined as a cut-off to select the genes for network construction. Construction and enrichment analyses of networks were done using networkanalyst (Xia et al., 2015).

*Regulome analyses of CRC*

To this end, we retrieved 621 samples related to CRC from TCGA through Regulome Explorer (Uddin et al., 2011; Kannan et al., 2015). To unfold the regulatory effects of different omics levels on protein level as a final manifest of central dogma process, we investigated the correlation between methylation, microRNA, point mutation, copy number alteration and gene expression profiles with protein levels in CRC. 

## Results

The total number of gene expression microarray samples for normal and cancer cells were 56 and 67, respectively (Sabates-Bellver et al., 2007; Uddin et al., 2011). Moreover, 1596 samples with point mutation data, 1354 samples with CNA data and 621 samples that applied in regulome analysis retrieved from cBioportal and TCGA (Table 1).

*Flux balance analysis reveals metabolic subsystems in CRC*

Impacted subsystems in colorectal cancer showed in Table 2. As it has been demonstrated in the supplementary file 3 in details, in the cancer metabolic model, the total number of 503 reactions had an increment in their flux values whereas 560 reactions had lower flux rates compared to normal metabolic models. Note that we ignored values smaller than 1×10^-10^ in the results.

According to FBA results the highest flux rate drop was in retinol dehydrogenase reaction in the “Vitamin A Metabolism” subsystem with the value of -1875. The notable flux value of -1398 was calculated for bicarbonate transport which is part of the” Transport Extracellular” subsystem. The results showed that in the “Pyrimidine Catabolism” subsystem, Cytosine deaminase reaction flux decreased in colorectal cancerous cells with the value of -529. Also, Glutathione peroxidase reaction in the “Glutathione Metabolism” subsystem represented a reduction in the colorectal cancer model with the value of -439. There was also a decrement in the “Transport Mitochondrial” subsystem in which the value of ADP/ATP transporter reaction was -382. 

There was a reduction in the “Fatty acid elongation” subsystem with the value of -147, which was calculated for palmitoyl-CoA desaturase reaction. In the “Glutamate Metabolism” subsystem, there was a reduction in glutamine synthetase reaction with the value of -129. Moreover, two other subsystems (“Oxidative Phosphorylation” and “Galactose Metabolism”) decreased in colorectal cancer model for ATP synthase and UTP-glucose-1-phosphate uridylyl transferase reactions with the values of -95 and -80, respectively.

However, FBA results demonstrated some subsystems in cancerous model with an increment in their reactions. For example, in the “Nucleotides” and “Pyruvate Metabolism” subsystems, there were increment with the value of 280 and 142, respectively which were due to nucleoside-diphosphate kinase and L-lactate dehydrogenase pathways. Also, Adenosine deaminase as a reaction in the “Purine Catabolism” subsystem showed increment in the colorectal cancer model with the value of 56. In” Glycolysis/Gluconeogenesis” subsystem, the highest flux value was for glucose-6-phosphate isomerase pathway with the value of about 53. Moreover, our results presented that hyaluronan synthase reaction with the value of 21 was increased in the “Hyaluronan Metabolism” subsystem.

*Differentially expression genes, point mutations and CNAs*

**Figure 1:**
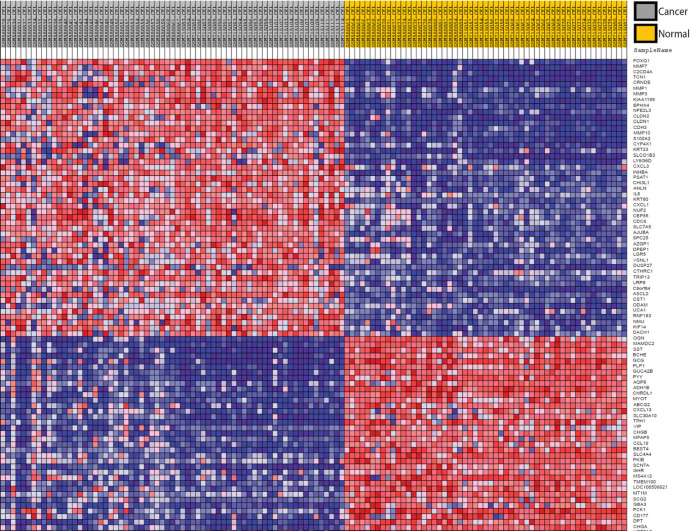
Differentially Expressed Genes in CRC Cells. Heatmap of 100 top ranked gene with altered expression

Differentially expression gene analysis shows the CRC gene signature including 396 genes with signal/noise ratio more than 0.5 and FDR<0.05. Figure 1 shows the heatmap of 100 top-ranked genes that altered in CRC cells. 

**Figure 2:**
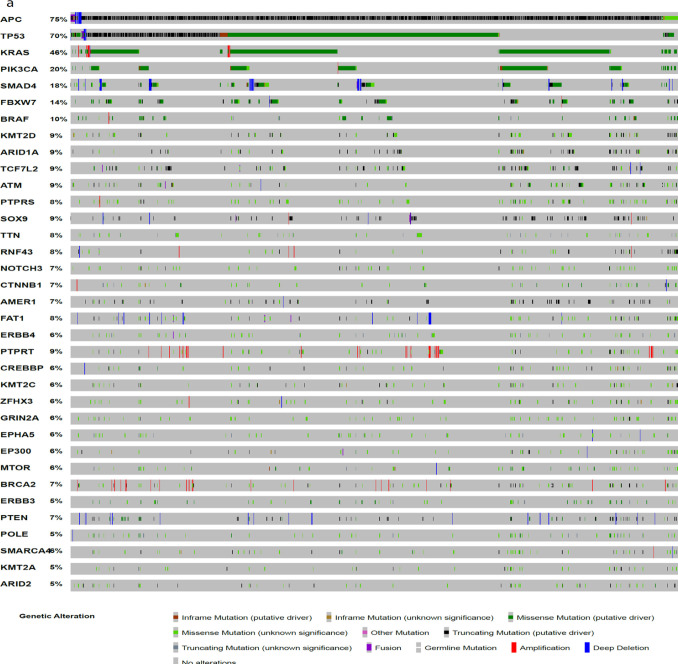
Point Mutation and Copy Number Aberration Hallmarks of CRC. (a) Heatmap of point mutations and, (b) CNAs show the genomic signatures related to colorectal cancer

Genomic data of 1,596 samples with point mutation data and 1,354 samples with CNA data retrieved from eight studies through cBioportal to find point mutation and CNA signatures corresponding to colorectal cancer. Analyzing the retrieved data indicated 7 genes with CNA (gain or lose area) and 37 genes with point mutations. Figure 2 shows the heatmaps of CNAs and point mutations related to CRC. 

*Pathway enrichment analysis results*

**Figure 3:**
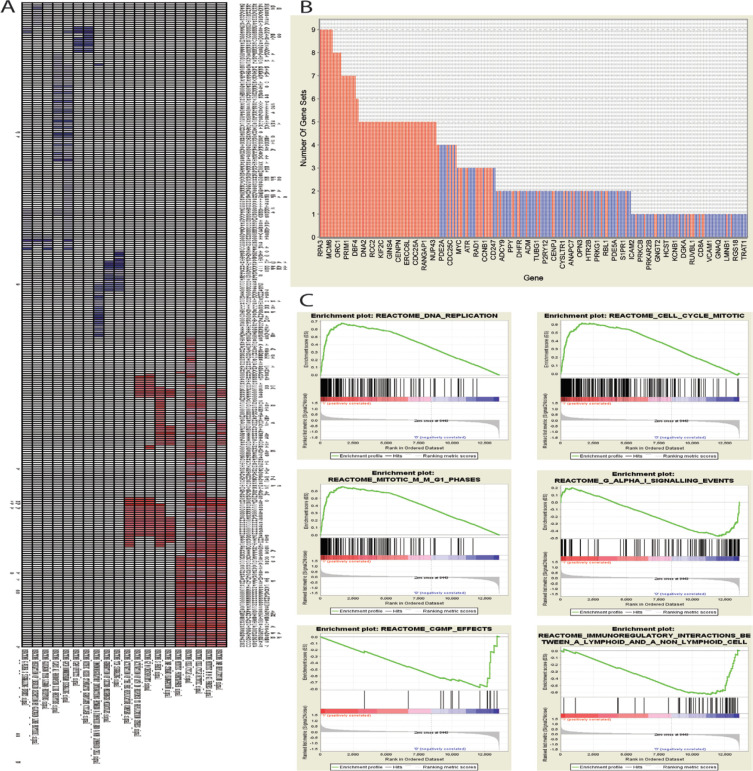
Gene Set Enrichment Analysis (GSEA) Results Show Impacted Pathway in CRC Cells. (a), Leading edge analysis results of 10 top overexpressed and 10 tops under expressed pathways that represented by a clustergram. (b), shows each enriched gene and the number of subsets in which it appears. (c), graphical view of the enrichment score of top three over and under expressed pathways that represented by GSEA plot. Peak of GSEA plot shows enrichment score for the gene set (FDR<0.05).

Enrichment analysis results revealed pathways that contributed to CRC phenotype. Some of the most impacted pathways were related to cell cycle and involved pathways with cell growth that represent as up-regulated pathways and conversely some pathways related to the immune system, rhodopsin-like receptors (class A/1) that serve as components of hormones, light, and neurotransmitter receptors are examples of down-regulated pathways in CRC cells. Figure 3 and Table 3 represent top enriched pathways. All enriched pathways are also available in the supplementary file 4 and 5.

*Tissue-specific network analysis*

**Figure 4:**
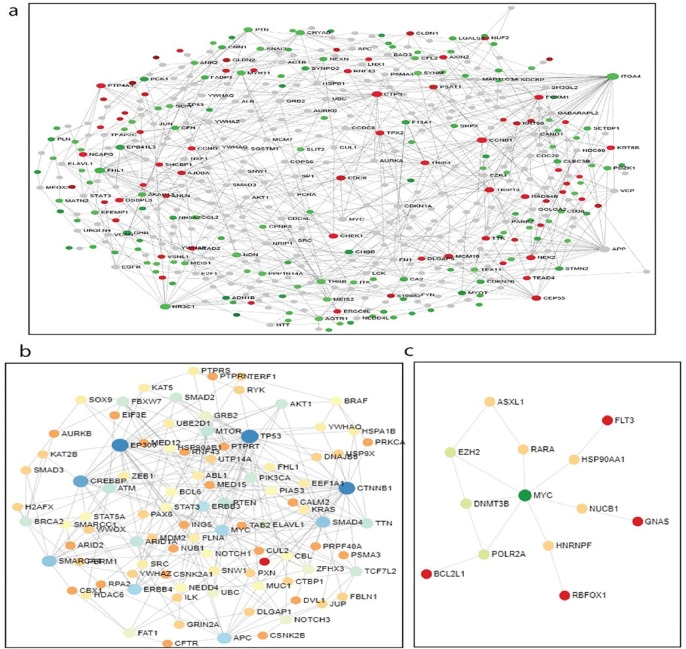
Constructed Networks of Genomic and Transcriptomic Signatures of CRC. (a) Network of DEGs that illustrate transcriptomic driver nodes in CRC. (b and c) PPIN of CRC that indicated nodes that affected by point mutations and CNA profile of CRC

**Figure 5:**
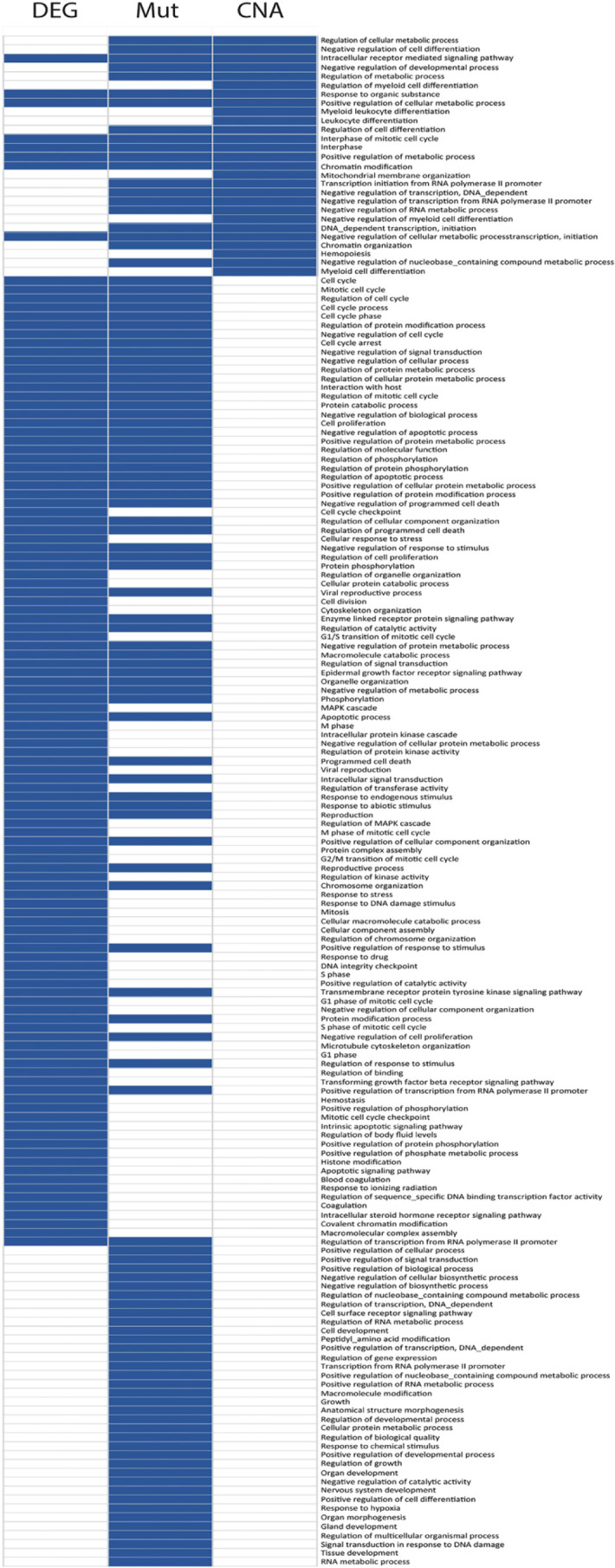
Clustergram of Network Enrichment Analysis. GO (BP) enriched terms by DEGs, point mutations and CNAs networks. This figure provides a snapshot from all biological process that affected in CRC

Analysis of three different networks constructed for DEG, point mutation and CNA signatures show different driver nodes related to CRC. The results show that finding driver nodes should be investigated in different level of omics network to obtain high reliable centrality for hub nodes of CRC. The result of PPIN of DEGs shows although this network illustrates a part of affected driver nodes of CRC, some CRC hallmarks cannot be found in this network. For example, in PPIN constructed by DEG, the P53 is not indicated as a driver node, while in point mutation network it was the most important node in the network. As a result, to find the CRC reliable hub nodes, applying comprehensive information from all of the omics levels is important. Figure 4 shows affected PPI networks for DEG, point mutation and CNA signatures. Also, the GO biological process enrichment analysis of networks created by genomic and transcriptomic signatures that mapped on tissue-specific protein-protein interaction network provides a perspective from all biological process underlying CRC (Figure 5). The most important hub nodes in each network presented in Table 4.

*Regulome analyses results*

**Figure 6:**
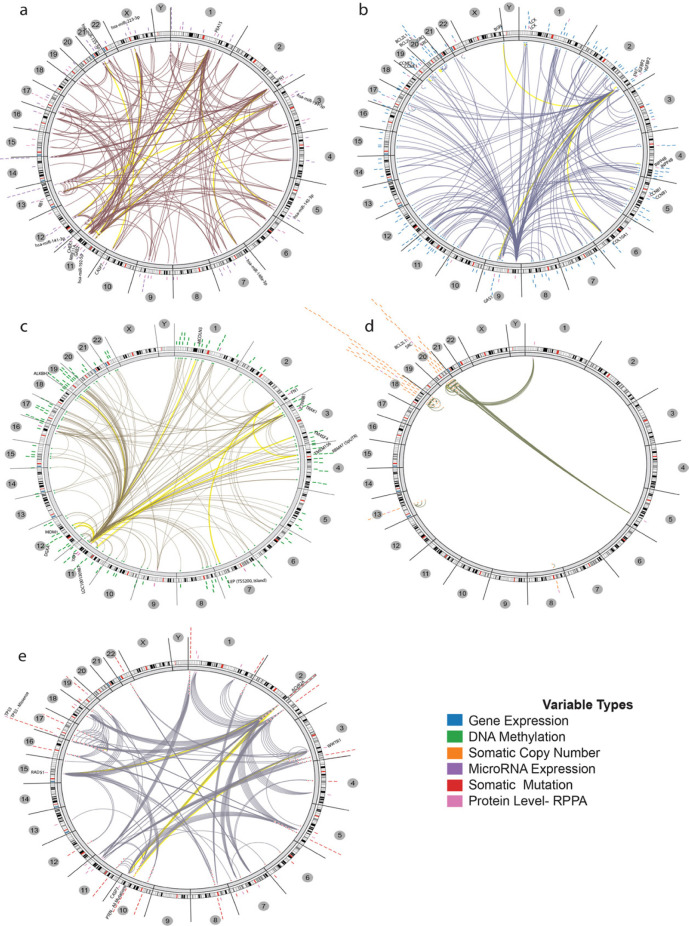
Regulome Scheme of CRC. This figure represents correlation of protein levels with the (a) microRNAs expression, (b) gene expression, (c) methylations, (d) copy number aberrations and (e) somatic point mutations in CRC. Yellow lines show top regulome interactions

Role of microRNA profile on protein level in CRC screened and indicated negative correlation of hsa-miR-148a-3p and hsa-miR-192-5p with FN1, a positive correlation of hsa-miR-223-3p and CHEK1, and a positive correlation of hsa-miR-155-5p and CASP7 as the most important microRNAs regulatory modules in CRC regulome, (Figure 6a and supplementary file 6). Also, considering correlation results between gene expression and protein levels show a positive correlation between *IGFBP2, BCL2L1, INPP4B* and *CCNE1* gene expression with their corresponding protein levels. Furthermore, COL10A1 gene expression level has a positive correlation with FN1 protein level, (Figure 6b and supplementary file 7). The regulatory effect analysis of methylation profile in CRC shows a positive correlation between methylation in 5pUTR of RBM47, DGKA, ALKBH7 and TRAK1, and YAP1 protein level, negative correlation between LOC100130987 methylation and CTNNB1 protein level and positive correlation of TMEM156 methylation with FN1 protein level (Figure 6c and supplementary file 8). The results of correlation between CNAs and protein level in the regulome framework suggest that CNAs have a low level of impact on the regulatory operating system of tumor cells in CRC to regulate protein level. But the most important of these regulatory interactions indicated for a positive correlation between chr20q12, 20q11, chr20q11 with BCL2L1 protein level as the most important correlations (figure 6d and supplementary file 9). Finally, considering the correlation results between point mutations and protein levels in CRC, TP53-missense has a positive correlation with TP53 level, PTEN-All mutations have a positive correlation with WWTR1, ACVR2A-all mutations have a positive correlation with CASP7, and ACVR2A-all mutations have a positive correlation with RAD51, (Figure 6e and supplementary file 10). 

## Discussion

Identification of altered agents in omics levels that are causally implicated in malignancy has been an overriding goal in understanding the cancer phenomenon. The growing body of high-throughput data coupled with the development of analyzing tools provided an opportunity for deciphering malignancy drivers and signaling pathways involved in tumorigenesis (Gao et al., 2013). Application of flux balance analysis in cancer is rapidly developing into a considerable scientific field and has been noticed as a helpful method for cancer diagnosis (Schulze and Harris, 2012). Indeed, the feasibility of data mining and exploration of subsystem fluxes provided a reliable explanation of metabolism alteration during cancer development (Schulze and Harris, 2012; Uhlen et al., 2017). In this study, we proposed a new metabolic model which is useful for cancer diagnosis and treatment. The results showed miscellaneous metabolic pathways in which flux changes were associated with the mechanism of cancer. According to FBA results, the highest reduction in flux levels for the CRC samples was in the “Extracellular Transport” subsystem. Such decrement has been discussed by Netti et al., (2000) which is caused due to cellular transfer reductions in the extracellular matrix (ECM). There is a controlling mechanism for molecular traffics in ECM. Barriers in molecular transport in ECM play an important role in tumor cells viability, for example, to prevent penetration of some therapeutic agents (Netti et al., 2000). Moreover, initiation of pro- or anti-apoptotic effects in cells particularly are related to ECM. Some of these functional components in cells promote tumor progression (Mott and Werb, 2004). Also, flux value for the “Vitamin A Metabolism” reduced in colorectal cancer cells. This result was in concordance with a previous study indicated the decrease of retinoic acid production consequently results in tumor immune evasion in colorectal cancer (Huynh et al., 2013). In addition to the FBA result that shows the reduction in the production of retinoic acid (which leads to tumor immune evasion), enrichment analysis of CRC’s DEG signature shows the “immune response” and “Class_A1_Rhedopsin_like_receptors” as examples of under-expressed pathways that were in concordance with the FBA results. Moreover, FBA results showed the Nucleotides Metabolism as an activated subsystem that is in concordance with the GSEA pathway analysis results (including activation of cell growth and involved pathways), (Table 2, 3 and Figure 3). Major alterations in energy metabolism occurred in cancerous cells. Mitochondria is the center for these changes in which the Warburg e-ect provides pyruvate for fermentation and oxidative phosphorylation process (Vander Heiden et al., 2009). Also, amino acid and lipid biosynthesis are the other biochemical pathways run through mitochondria (Rizzuto et al., 2012; Andalib et al., 2013). It is expected when cells become cancerous, changes occur in biochemical transportation of cancer cells (Lytovchenko and Kunji, 2017). It has been previously confirmed that because of the increment in glycolysis and lactate pathways in tumors, the suitable condition would be achieved for tumor growth (Solaini et al., 2011). In this study, the FBA results explicitly represented a high level of flux response in the “Glycolysis” subsystem as it was expected. Previous studies also demonstrated lipid metabolism alterations in colon tumors (Keshk et al., 2014), and fatty acid elongation may serve as a therapeutic and detection marker in colorectal cancer (Yan et al., 2016). FBA results showed a reduction in the “Fatty acid elongation” subsystem. Moreover, former studies underscored the role of hyaluronidases in colorectal cancer. Indeed, tissue distribution of hyaluronidase reaction and the concept of certain isoforms in each tumor stages indicated a new evidence of involving in the mechanism of colorectal cancer progression (Bouga et al., 2010). Analyzing the genomic data including point mutation and CNA data also revealed signatures related to CRC that could not be found by analyzing single omics level. As an example, P53 that plays a role as a cancer hallmark was not detected in transcriptome analysis. Hence, the findings of this study show that it is necessary to study cancer as a systemic and complicated disease through all omics levels simultaneously. Figures 1, 2 and 4 and Table 4 show different signatures in gene expression, point mutation and copy number alteration profile of CRC. Among these signatures, Adenomatous Polyposis Coli (APC) burdens high frequency of inactivating mutations (76%) in 1,596 analyzed samples (these mutations mostly included truncated mutations and deep deletions) and it serves as an early driver for CRC (Genomic map in Figure 2). One of the most important driver pathways in CRC is an abnormality of the WNT signaling pathway which extensively studied in relation with APC mutations (Müller et al., 2016). Moreover, the driver role of the APC represented in the network which indicated as a hub node in different functional modules. Moreover, KRAS, TP53, and SMAD4 are the other hallmarks of CRC that serve important roles in progression of CRC. Their functional roles in PPINs were highlighted in the enrichment results of networks (Figure 5). The obtained PPINs from mapping transcriptomic and genomic signatures also showed different functional network modules (Figures 4 and 5). These different cancerous network modules promote cancer in the framework of interconnected omics interactions. The goal of pan-omics in cancer studies is to identify driver agents that may be useful for finding diagnostic, prognostic and other markers related to different outcomes of disease. Screening in the result of regulome section of this study showed correlation between PTEN mutation and WWTR1 protein level (figure 6e and supplementary file 10). This result also observed in a previous study that indicated the WWTR1 protein level was high in cancer cells with up-regulated PI3K signaling in PTEN mutant tumor cells (Huang et al., 2012). A literature search was done to find a correlation between WWTR1 mutation and CASP7 protein level that indicated in our results (Figure 6e and supp file 10), but we could not find a correlation between WWTR1mutation and CASP7 protein level in previous studies. As reported previously, ACVR2A harboring genomic instability in CRC cells and was correlated with RAD51 that involves in DNA damage repair process (Brough et al., 2012; Kim et al., 2013). Subsequently, the correlation between ACVR2A mutations and RAD51 protein level indicated in regulome results of this study. Analyzing the methylation effect on regulome network of CRC revealed the significant regulatory effect of RBM47 methylation over YAP1 protein level. Since YAP1 is a key component of Epithelial–Mesenchymal Transition (EMT) regulation (Fisher et al., 1994), the results suggest that RBM47 contributes to EMT through regulating YAP1 level in CRC that may trigger metastasis process (De Craene and Berx, 2013). The results highlighted YAP1 as a key node in methylation regulatory network that affected by methylation of DGKA, TRAK1, ALKBH7, TM4SF4, MCOLN3, MDM1 and a long list of genes that represented in supplementary file 8. Evaluating the regulatory effect of micro-RNA profile of CRC on protein levels indicated correlation of hsa-miR-148a-3p with FN1, correlation of hsa-miR-155-5p with CASP7 and correlation of hsa-miR-143-3p with PEA15 (Figure 6 and supplementary File 6). This result was in concordance with the previous study that profiled microRNA expression and regulatory effect of top-ranked microRNA in CRC (Kara et al., 2015). While data mining of one specific omics data is a promising approach to find predictive signatures of different biological states, the promise will be limited if findings are not considered the interaction of omics levels in an interconnected biological network. Findings of this study likely would help the future studies to understand molecular phenomena of colorectal cancer. 

In conclusion, considering paradigm shift of cancer studies that focus on the personalized study of cancers which ignore the heterogeneity of tumors and also paucity of multi-omics studies of colorectal cancer, this study directed to provide a reliable population-based perspective from molecular mechanisms underlying CRC. These results obtained from analysis of huge and different types of data to uncover alterations that are difficult to explain by analysis of one omics level in CRC. 

**Table 1:** Summary of CRC Data Used in This Study

Gene expression microarray data	
Source	Number of samples
GSE8671	64 (32 normal, 32 cancer)
GSE23878	59 (24 normal, 35 cancer)
Copy number aberration data	
Source	Number of samples
Cbioportal	1354
Point mutation data	
Source	Number of samples
Cbioportal	1596
Data applied for regulome analyses	
Source	Number of samples
TCGA	621

**Table 2:** Altered Reactions in the Subsystems for Colorectal Cancerous Model Compared to the Normal Model

Altered Subsystem	Altered Reaction	Alteration type (Tumor Model)
Vitamin A Metabolism	Retinol Dehydrogenase	Decreased
Transport Extracellular	Bicarbonate Transport	Decreased
Pyrimidine Catabolism	Cytosine Deaminase	Decreased
Glutathione Metabolism	Glutathione Peroxidase	Decreased
Transport Mitochondrial	ADP/ATP Transporter	Decreased
Fatty Acid Elongation	Fatty Acyl-CoA Desaturase	Decreased
Glutamate Metabolism	Glutamine Synthetase	Decreased
Oxidative Phosphorylation	ATP Synthase	Decreased
Galactose Metabolism	UTP-Glucose-1-Phosphate Uridylyltransferase	Decreased
Nucleotides Metabolism	Nucleoside-Diphosphate Kinase	Increased
Pyruvate Metabolism	L-Lactate Dehydrogenase	Increased
Purine Metabolism	Adenosine Deaminase	Increased
Glycolysis/Gluconeogenesis	Glucose-6-Phosphate Isomerase	Increased
Hyaluronan Metabolism	Hyaluronan Synthase	Increased

**Table 3:** The most Impacted Pathways that Enriched by Reactom Geneset

Under-expressed pathways		Over expressed pathways	
NAME	FDR (q-val)	NAME	FDR q-val
Reactome_immunoregulatory_interactions_between_a_lymphoid_and_a_non_lymphoid_cell	0	Reactome_dna_replication	0
Reactome_g_alpha_s_signalling_events	0.000434	Reactome_mitotic_m_m_g1_phases	0
Reactome_cgmp_effects	0.002634	Reactome_cell_cycle_mitotic	0
Reactome_class_a1_rhodopsin_like_receptors	0.002183	Reactome_cell_cycle	0
Reactome_gpcr_downstream_signaling	0.001746	Reactome_g2_m_checkpoints	0
Reactome_nitric_oxide_stimulates_guanylate_cyclase	0.001455	Reactome_mitotic_prometaphase	0
Reactome_generation_of_second_messenger_molecules	0.00283	Reactome_activation_of_atr_in_response_to_replication_stress	0
Reactome_tcr_signaling	0.003662	Reactome_dna_strand_elongation	0
Reactome_gpcr_ligand_binding	0.003533	Reactome_s_phase	0
Reactome_glucagon_type_ligand_receptors	0.003693	Reactome_activation_of_the_pre_replicative_complex	0

**Table 4:** The Most Important Hub Nodes in Network Created by Transcriptomic and Genomic Signatures

DEGs network			Point mutation network			CNA network		
Label	Degree	Betweenness	Label	Degree	Betweenness	Label	Degree	Betweenness
ITGA4	402	819295.4	TP53	556	596332.47	MYC	849	492887.81
TRIP13	75	146383.85	EP300	293	252816.94	BCL2L1	66	54377.5
PTP4A3	73	138377.42	CREBBP	196	127599.6	DNMT3B	35	28506.19
CCNB1	57	127533.74	CTNNB1	194	182161.38	RBFOX1	29	25624.5
NR3C1	54	159978.78	SMARCA4	100	93783.27	GNAS	28	21732.5
PTN	49	79172.5	SMAD4	100	78005.5	ASXL1	11	5978
THRB	46	89620.76	APC	89	96969.15	FLT3	8	5973.5
EPB41L3	45	94762.82	FBXW7	82	85781.6	POLR2A	3	6998.88
PCK1	45	60748.27	MTOR	68	59152.88	EZH2	3	1577.2
